# Annual Meeting of the Esculapian Society

**Published:** 1857-12

**Authors:** C. M. Hamilton, D. W. Stormont

**Affiliations:** President; Secretary


### PROCEEDINGS OF MEDICAL SOCIETIES.

### PROCEEDINGS OF THE ANNUAL MEETING OF THE ESCULAPIAN SOCIETY, HELD IN PARIS, ILL., OCT. 28TH & 29TH.

The Esculapian Society met in the Christian Church, in Paris, Oct. 28th, 1857, and was called to order at 1 o'clock. Twenty-five members were present. The minutes of the last meeting were read by the Secretary, and approved.

Drs. Hamilton and Johnson were appointed to fill vacancies in the Board of Censors.

Dr. McCord, the Treasurer, submitted his annual report. On motion of Dr. Chambers, it was received and ordered to be filed.

Dr. F. R. Payne, the President, delivered his valedictory address on the Powers of Nature in curing disease.

Reports from the Standing Committees were called for, but there were none ready.

Essays and reports of cases called.

Dr. Stormont read a paper on the cure of Acute and Sub-acute Rheumatism, by seductive doses of tartarized antimony, which was discussed by several members.

Dr. S. York read an excellent essay on Granular Conjunctivitis. Discussed by Drs. McCord, Payne, Chambers, Johnson, Davis, Hamilton, Goodhart, Clippinger and York.

On motion, the Society adjourned to meet at 7 o'clock.

### EVENING SESSION.

The Society met, according to adjournment, the President, Dr. Payne, in the chair.

Dr. Hinkle, being introduced, delivered the annual address, on the Elements of the True Physician, which was received with marked favor by a respectable audience.

Dr. Chambers moved the thanks of the Society be tendered Dr. Hinkle, for his address, and a copy requested for publication. Carried.

After the dismissal of the audience, on motion of Dr. McCord, the Society went into the election of officers for the ensuing year, which resulted as follows:

| | |
|---|---|
| Dr. C. M. Hamilton, . . . . . . . | President. |
| " H. W. Davis, . . . . . . . . | Vice-President. |
| " D. W. Stormont, . . . . . . . | Secretary. |
| " J. Ten Brook, . . . . . . . . | Treasurer. |

Drs. McCord, York, Herrick, Hinkle and McAllister—Censors.

Drs. York, Chambers and Hinkle were appointed a Business Committee, to report to-morrow morning.

Dr. Chambers read an excellent paper on Inflammation, Induration and Ulceration of the Os and Cervix Uteri, with the use of the Speculum. Discussed by Drs. York, Clippinger, Davis and Chambers.

On motion, the Society adjourned to meet to-morrow morning, 8 o'clock.

THURSDAY, OCT. 29TH.—MORNING SESSION.

The Society met, as per adjournment, Dr. Hamilton in the chair.

On motion of Dr. York, Dr. Kile, who was present, was invited to participate in the discussions.

Dr. Bowers reported a case of Dysentery. Discussed by several members.

The President appointed the following gentlemen Chairmen of the Standing Committees, to report on the subjects annexed to their names, at the next *annual* meeting, viz:

Dr. S. York—Surgery.
" D. O. McCord—Practical Medicine.
" D. W. Stormont—Midwifery.
" F. R. Payne—Epidemics.
" B. F. Swafford—Indigenous Botany.

The Business Committee reported the following subjects, and the gentlemen, whose names are opposite, were appointed to write thereon, for the next meeting, viz:

Hooping Cough—Dr. Smith.
Uterine Tumors—Dr. Chambers.
Sequences of Intermittent Fever—Dr. Bowers.
Permanent Cure of Reducible Hernia—Dr. Swafford.
Gonorrhea—Dr. Willits.
Delirium Tremens—Dr. Davis.
Epilepsy—Dr. F. R. Payne.
Milk-Sickness—Dr. J. W. York.
Ovariotomy—Dr. Goodhart.
Tuberculosis—Dr. Stormont.
Spinal Irritation—Dr. Frizell.
Scrofulous Ophthalmia—Dr. McAlmont.
Diptheritis—Dr. S. York.
Inflammation of Internal Ear—Dr. H. R. Payne.
Menorrhagia—Dr. Apperson.
Inflammation and Hypertrophy of the Tonsil Glands—Dr. Mitchel.
Appearance of the Tongue as an Indication of Disease—Dr. Herrick.

Vomiting in Pregnancy—Dr. Ten Brook.

Croup—Dr. McCord.

The Causes which have brought about the necessity of a change in the General Treatment of Diseases from a Depletory to a Stimulating—Dr. Hinkle.

On motion of Dr. Stormont, the Society went into an election for delegates to the American Medical Association and to the State Medical Society, which resulted as follows:

To the National Association—Drs. Hamilton, Steele, S. York, McCord and Hinkle.

To the State Society—Drs. S. York, J. M. Steele, H. R. Payne, H. W. Davis and J. W. Yorke.

On motion of Dr. Stormont, each delegate was authorized to appoint a substitute in case he cannot attend.

On motion of Dr. Chambers, the delegates to the State Society were instructed to vote for the adoption of a resolution condemning the "hireling system" as derogatory to the profession.

Dr. Chambers introduced to the Society a gentleman with extensive caries of the femur, of fifteen years' standing, which he proposed to remove by excision. The case was examined with much interest by the members, and the merits of this operation discussed by Drs. Kile, Hinkle, Davis and Chambers.

Dr. Smith read an essay on Variola, and reported a case of Eclampsia. Both papers were extensively discussed.

On motion, the Society adjourned to meet at 1 o'clock.

AFTERNOON SESSION.

The Society met, the President in the chair.

Dr. Davis read an excellent essay on Pneumonia. Discussed by Drs. York and Chambers.

Dr. E. Read, of Terre Haute, an honorary member of the Society, by invitation, read a lengthy paper on the changes that have occurred in medical practice and the causes thereof, which was listened to with much interest.

On motion of Dr. York, the thanks of the Society were tendered Dr. Read, and a copy of his paper requested for publication.

Marshall was selected as the place for holding the next meeting.

Dr. Herrick was appointed to deliver the next public address.

Drs. H. R. Payne, C. Duncan and D. O. McCord were appointed the Committee of Arrangements.

Dr. McCord offered the following resolution:

*Resolved*, That the Transactions of this Society be published annually, at the cost of the Society, commencing one year from this date.

On motion of Dr. Stormont, this resolution was laid on the table until the next meeting.

Dr. Stormont moved the thanks of the Society be tendered the Christian Church, in this place, for the use of their house. Carried.

Dr. McCord moved that the thanks of the Society be tendered the physicians of Paris for their hospitality on this occasion. Carried.

During the meeting, Drs. J. W. York, of Windsor; G. A. Goodhart, of Hutsonville; H. C. McAllister, of Elkton; B. F. Swafford, of Paris; and A. S. Stevens, of Casey, were elected members of the Society.

Dr. S. J. Young, of Terre Haute, was elected an honorary member.

A copy of these proceedings was ordered to be furnished the *N. W. Medical and Surgical Journal* for publication.

The Society then adjourned to meet in Marshall on the last Wednesday in May next.

C. M. HAMILTON, *President*.

D. W. STORMONT, *Secretary*.

